# An ancestral hard-shelled sea turtle with a mosaic of soft skin and scutes

**DOI:** 10.1038/s41598-022-26941-1

**Published:** 2022-12-31

**Authors:** Randolph Glenn De La Garza, Henrik Madsen, Peter Sjövall, Frank Osbӕck, Wenxia Zheng, Martin Jarenmark, Mary H. Schweitzer, Anders Engdahl, Per Uvdal, Mats E. Eriksson, Johan Lindgren

**Affiliations:** 1 Department of Geology, Lund University, Lund, Sweden; 2 Mo-clay Museum, Nykøbing Mors, Denmark; 3 Materials and Production, RISE Research Institutes of Sweden, Borås, Sweden; 4 Museum Salling, Fur Museum, Skive, Denmark; 5 Department of Biological Sciences, North Carolina State University, Raleigh, NC USA; 6 Medical Microspectroscopy, Biomedical Center, Lund University, Lund, Sweden; 7 Department of Chemistry, Lund University, Lund, Sweden; 8 North Carolina Museum of Natural Sciences, Raleigh, NC USA

**Keywords:** Palaeontology, Herpetology, Cell biology, Molecular biology

## Abstract

The transition from terrestrial to marine environments by secondarily aquatic tetrapods necessitates a suite of adaptive changes associated with life in the sea, e.g., the scaleless skin in adult individuals of the extant leatherback turtle. A partial, yet exceptionally preserved hard-shelled (Pan-Cheloniidae) sea turtle with extensive soft-tissue remains, including epidermal scutes and a virtually complete flipper outline, was recently recovered from the Eocene Fur Formation of Denmark. Examination of the fossilized limb tissue revealed an originally soft, wrinkly skin devoid of scales, together with organic residues that contain remnant eumelanin pigment and inferred epidermal transformation products. Notably, this stem cheloniid—unlike its scaly living descendants—combined scaleless limbs with a bony carapace covered in scutes. Our findings show that the adaptive transition to neritic waters by the ancestral pan-chelonioids was more complex than hitherto appreciated, and included at least one evolutionary lineage with a mosaic of integumental features not seen in any living turtle.

## Introduction

The development of a squamous integumental covering is a major evolutionary innovation that enabled colonization of terrestrial habitats by sauropsids (that is, reptiles, birds and their extinct ancestors)^1^. Not to be confused with the dermally derived scales of bony fish^2^, reptilian scales comprise plates of folded epidermis with cells that contain both α-keratin and corneous beta proteins (CβPs; formerly referred to as β-keratin)^1–3^. Inclusion of the lineage-specific CβPs reinforces the reptilian integument, thereby providing a tough physiochemical barrier against external stressors, such as mechanical abrasion and pathogens^1–4^, while simultaneously maintaining homeostasis by preventing dehydration^1,2^.

Turtles (Testudines) are a diverse group of sauropsids renowned for their conspicuous shells. Externally, the carapace and plastron are covered by large, CβP-rich horny shields termed scutes^2,5^, while scales also protect the head, lower limbs and feet^2,5^. However, in most extant species, the neck, upper extremities and tail are enveloped by an epidermis with a greater proportion of α-keratin, leading to a more flexible skin in these areas^6^. This integumental mosaic of hard scales and softer skin is common among living turtles^5^, to suggest that it represents the basal condition for testudines^7^. Nevertheless, exceptions do exist, and these, in part at least, are thought to be related to either habitat choice or lifestyle^8,9^. Obligate aquatic turtles, such as members of the Dermochelyidae, Carettochelyidae and Trionychidae, are noteworthy for their reduced scalations. Adult dermochelyids even lack these integumental appendages, with juveniles losing scales in favor of a smooth, leathery skin during their ontogenetic transition to adulthood^9–11^. Members of the Carettochelyidae and Trionychidae independently evolved partial scale reduction, losing scutes and the majority of their body scales in the process^12^. However, these turtles retain scales in a few places, with carettochelyids possessing them, e.g., along the leading edge of their flippers^11,13^. The secondary loss of scales in dermochelyids, carettochelyids and trionychids seems to have occurred in tandem with both shell reduction and the development of specialized hydrodynamic limbs^14,15^.

On the other hand, hard-shelled sea turtles (Cheloniidae), despite being closely related to dermochelyids^16^, retain heavy scalations throughout their lives, with most extant species having large, sub-rectangular scutes on the shell and polygonal to rectangular scales on the limbs^10^.

**Figure 1:**
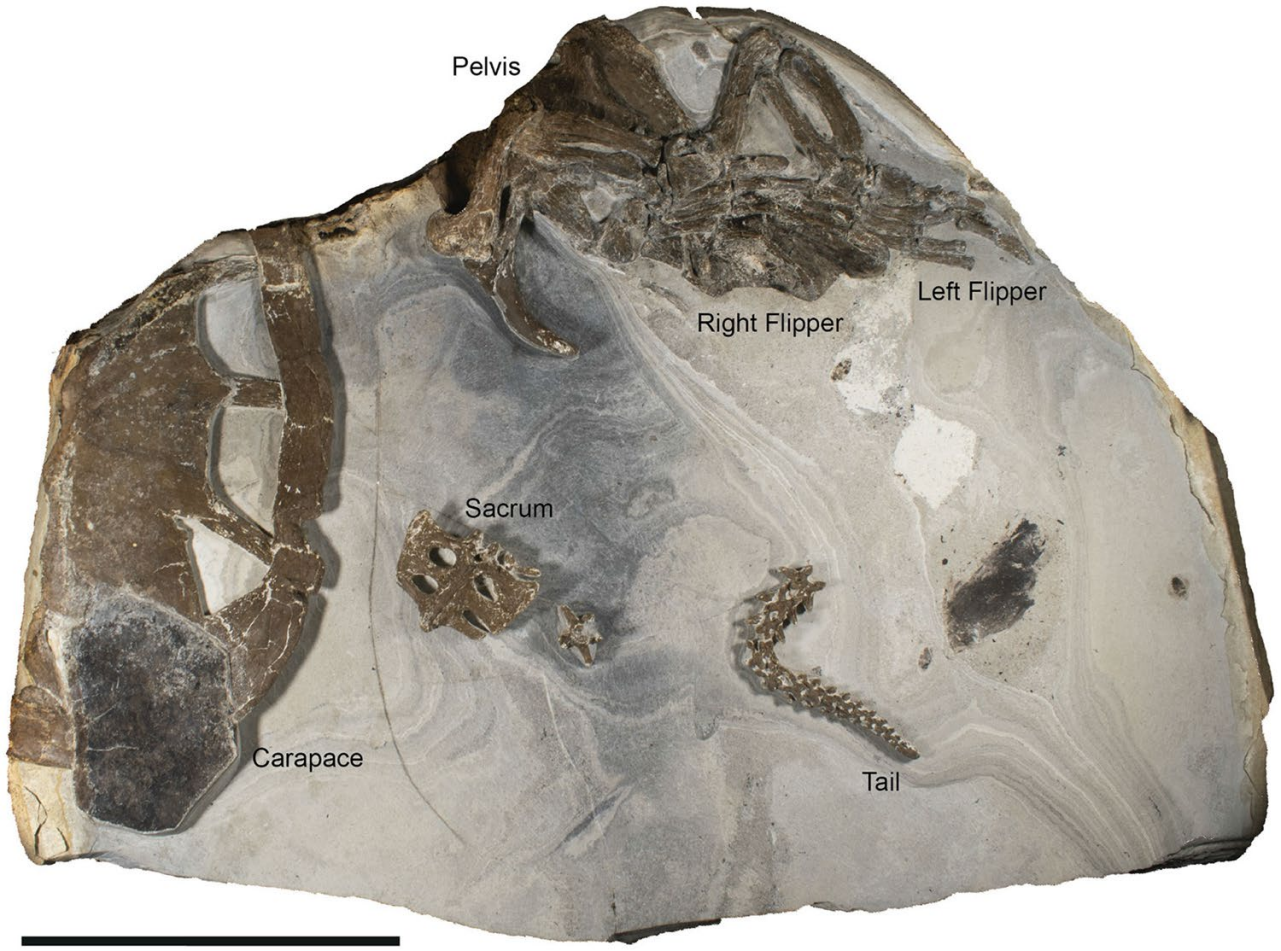
Photograph of DK 807, Pan-Cheloniidae indet., from the earliest Eocene Fur Formation of Denmark. Scale bar, 20 cm.

Fossils documenting the evolutionary reduction, and occasionally complete loss, of scales in soft-skinned turtle limbs are hitherto lacking. Nonetheless, the existence of epidermal scutes has been inferred in turtles as old as the Late Triassic (some 220 million years ago) based on scute sulci on the skeletal carapace and plastron^17^, and a progressive loss of scutes has been previously documented in carettochelyids^13^. Still, little is known about the actual scalation patterns of most extinct turtles. In part, this is because fossilized turtle integument is exceedingly rare, with only a handful of specimens showing remnant soft tissues (e.g., Refs.^18–20^; see also Supplementary Information). Recently, however, a partial but largely articulated sea turtle skeleton with associated soft tissues was reported by De La Garza et al.^21^ (Fig. 1). The fossil (DK 807; Mo-clay Museum, Nykøbing Mors, Denmark), from marine Eocene aged (about 54 million years ago) strata of the Fur Formation of Denmark, was considered to be closely related to the pan-cheloniid *Eochelone*^21^. While the previous study provided a general description of the fossil, focusing on skeletal features and inferred bite marks, the present contribution documents the detailed structural and biomolecular composition of the preserved soft parts.

## Results

### Geological context and age

The Fur Formation (locally ‘Mo-clay’) of the western Limfjord region, Denmark, is a clayey diatomite with interbedded carbonate concretions of earliest Eocene (Ypresian) age^22^. The mostly finely laminated sediments are thought to have accumulated below the storm wave base in a restricted marine basin at depths ranging between 100 and 500 m^22^. A large number of basaltic volcanic ash layers originating from eruptions in the nearby North Atlantic Igneous Province has facilitated precise local correlations of the sedimentary succession^22^, and the deposits have experienced minimal alteration due to low geothermobaric conditions^23^. As a consequence, the sediments of the Fur Formation preserve a unique faunal and floral assemblage of mixed terrestrial, coastal and pelagic components, including bony fishes, insects, reptiles, birds, and plants^22^. Collectively, these fossils reflect the organismal diversity immediately after the most pronounced greenhouse event of the Cenozoic (that is, the Paleocene–Eocene Thermal Maximum)^22^.

### The fossil

DK 807 was collected in 2013 from a single carbonate concretion, and subsequently prepared using a combination of mechanical tools and a 10% solution of acetic acid buffered with sodium acetate and calcium orthophosphate. The fossil comprises a partial carapace, pelvic girdle, intact hind limbs, sacrum, and a consecutive series of articulated caudal vertebrae; all derived from a single, moderate-sized sea turtle (Fig. 1)^21^. Some skeletal elements (e.g., the carapace and right hind limb) are exposed in dorsal aspect; others (e.g., the pelvis, sacrum and left hind limb) are seen in ventral view (Fig. 1). The individual bones retain their original three-dimensional shape, although some have suffered slight crushing from diagenetic compaction. While precise taxonomic identification has proved difficult (due to the incomplete nature of the fossil)^21^, the skeletal anatomy of DK 807 compares favourably with the extinct pan-cheloniid *Eochelone—*a genus of sea turtle with a wide geographic distribution during the Eocene^24–27^.

### Scutes and skin

Remnants of soft tissues are extensive in DK 807, and preserved as thin, bedding-parallel films of dark matter. A large sub-hexagonal scute covers part of the posterior segment of the bony carapace (Fig. 1 and Supplementary Fig. S1a). This epidermal appendage is similar in both shape and proportions to vertebral scutes of extant cheloniids, with its position at the edge of the shell indicating that the scute has moved somewhat from its original location during the decay of the carcass. A second, more incomplete scute is represented by a narrow strip of brownish material that partly shields costals 6 and 7^21^. Based on its location (and assuming limited post-burial displacement), this integumental residue likely represents the remains of a pleural scute.

**Figure 2:**
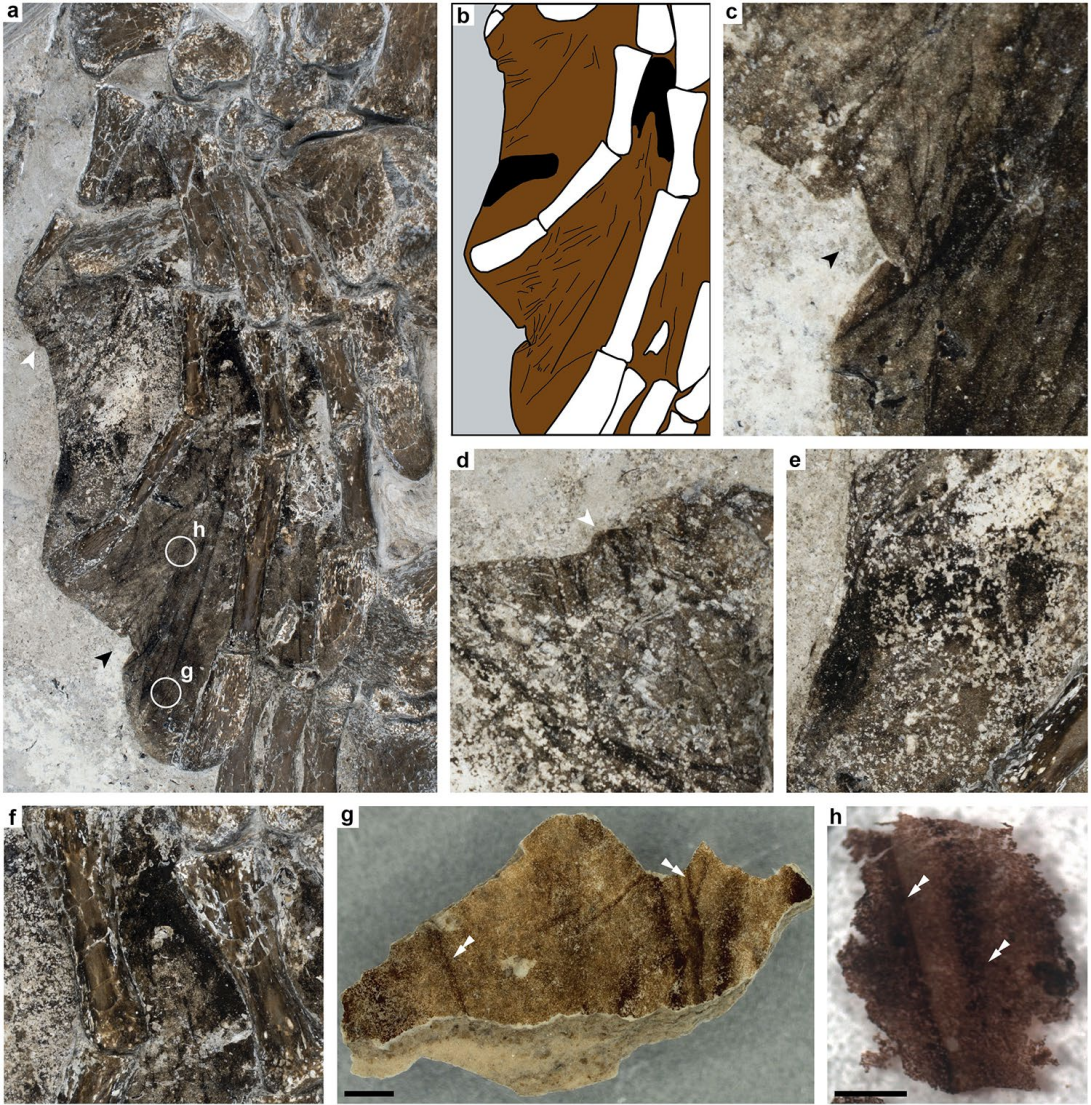
Structure of DK 807 hind limb soft tissues. (**a**) Photograph of the right hind flipper of DK 807. Approximate areas from where samples were taken from the counterslab for analysis are demarcated by white circles (**g**,**h**). (**b**) Sketch image of some key features of the flipper, with brown indicating epidermal tissues, grey sediment, white bone, and black dermal remains. Lineations are interpreted as folded tissue residues. (**c**,**d**) Close-up photographs of multiple skin folds. The locations of these folds are denoted by corresponding arrows in (**a**). (**e**,**f**) Patches of concentrated black matter within the flipper soft tissue, interpreted as dermal residues. (**g**) Image of sampled flipper residue demineralized using EDTA. (**h**) Photograph of a liberated sheet of organic matter following treatment with hydrofluoric acid. Note multiple lineations (folded tissue residues) denoted by white double arrowheads in (**g**) and (**h**). Scale bars, 1 mm (**g**,**h**).

The soft tissues of the hind limbs are mainly represented by a distinct black-brown halo that surrounds the pes of the right paddle (Figs. 1, 2a), although dark patches also occur in the left flipper (Fig. 1). The soft parts of the right limb are largely confined to spaces in between the metatarsals and phalanges, where they form a webbed structure that abuts digits II–V (Fig. 2a,b). Macroscopically, the preserved material is sheet-like (Fig. 2h), and visually distinct from the surrounding diatomaceous sedimentary matrix. Multiple darker lines run across the surface of the film, with the most prominent ones lying between digits III and IV (Fig. 2a,b). These are mostly straight and parallel to the digits, although some bifurcate into patterns of intersecting lines (Fig. 2b–d). In addition, small portions of the film appears to have folded on top of itself, revealing sections of the flipper that originally faced the underlying sediment (Fig. 2a,c,d, black and white arrows).

**Figure 3:**
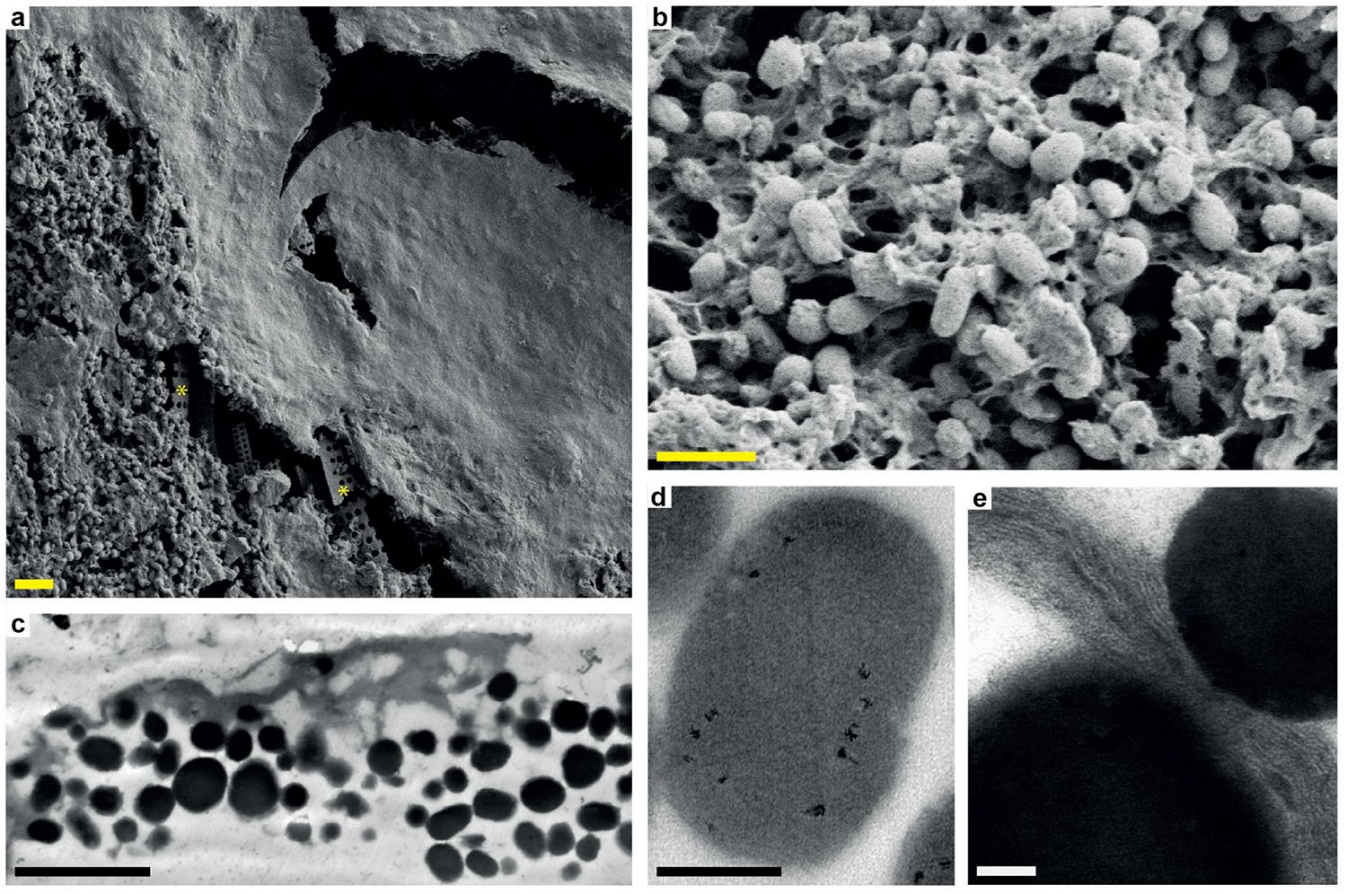
Ultrastructure of DK 807 hind limb soft tissues. (**a**) FEG-SEM micrograph of the skin residue with sheet-like matter covering amassed melanosomes. Diatom frustules (indicated by yellow asterisks) are visible underneath the organic matter. (**b**) Higher magnification FEG-SEM micrograph of melanosomes embedded in a vesicular matrix. (**c**) TEM micrograph of demineralized fossil flipper residue in cross-section. Note electron dense melanosomes covered by sheet-like matter. (**d**) High magnification TEM micrograph of a melanosome. (**e**) High magnification TEM micrograph of the matrix in between two melanosomes. Note filamentous structures arranged as stacked laminae. Scale bars, 2 μm (**a**,**c**), 1 μm (**b**), 200 nm (**d**), 100 nm (**e**).

At higher magnification, demineralized tissue samples from both the right hind limb (Fig. 2g,h) and one of the scutes (Supplementary Fig. S1b) were resolved as dense aggregations of spheroid to ovoid microbodies (Fig. 3a–d and Supplementary Fig. S1c–e); these appeared as solid objects under both FEG-SEM (Fig. 3a,b and Supplementary Fig. S1c) and TEM (Fig. 3c,d and Supplementary Fig. S1d,e). The microbodies occurred intertwined with a fibrous to spongious matrix that was particularly prominent in the flipper samples (Fig. 3a–c). In some areas, the vesicular matter was dense enough to form a semi-continuous, sheet-like structure that partly obscured the microbody-rich layer (Fig. 3a). When visualized under TEM, this coating came across as more electron-translucent than the microbodies (Fig. 3c,e). Moreover, stained sections revealed an internal composition comprising multiple stacked laminae with individual thicknesses of about 8 to 10 nm (Fig. 3e).

Sediments adjacent and directly underneath the soft tissues consisted mostly of clay particles and spheroid to rod-shaped diatom frustules (Fig. 3a, asterisks), with the latter intermittently perforating the microbody-rich film (Supplementary Fig. S1c).

### Elemental and chemical composition

**Figure 4:**
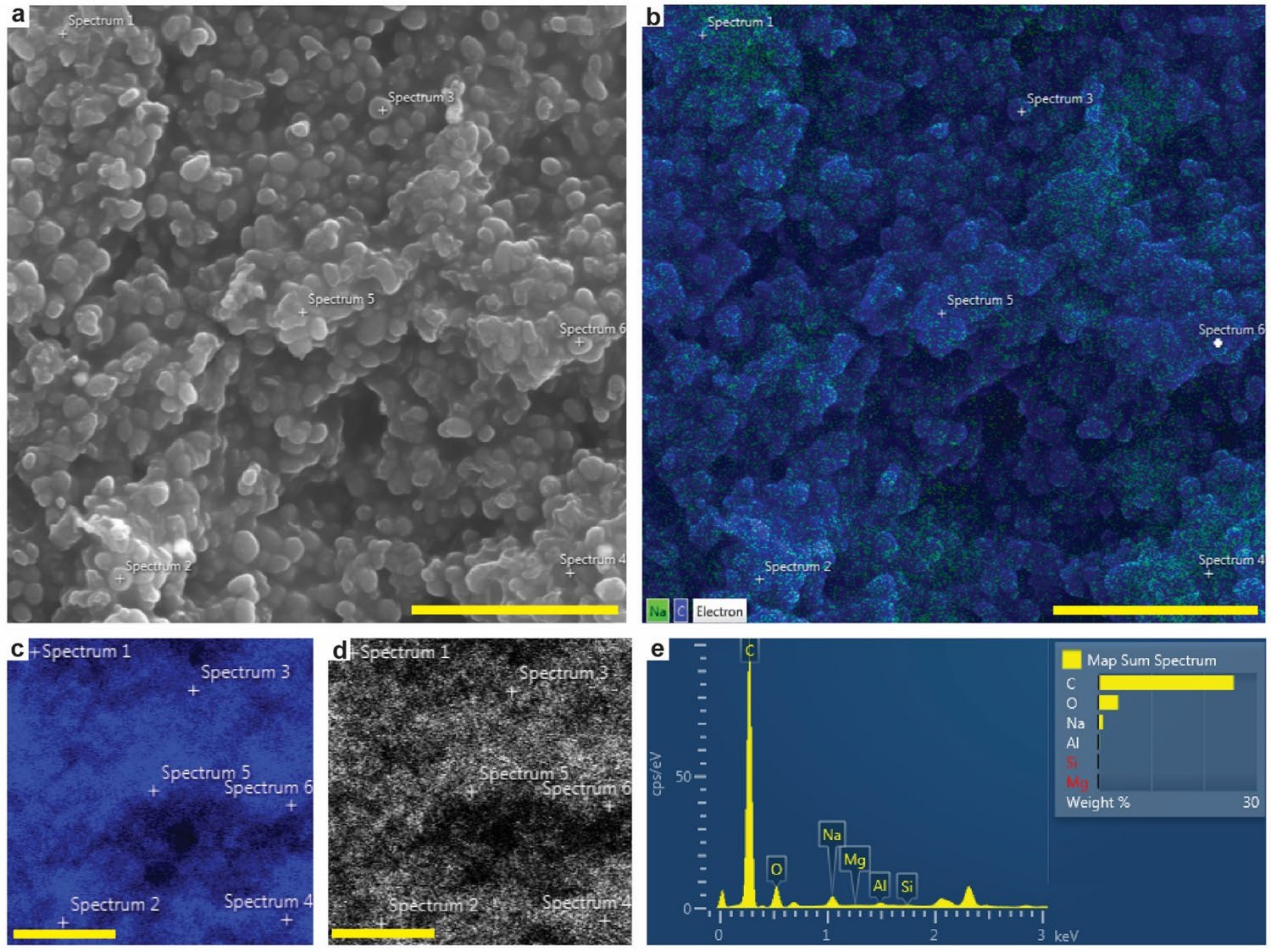
Energy dispersive X-ray microanalysis of HF demineralized fossil flipper tissue. (**a**) FEG-SEM micrograph showing abundant melanosomes. (**b**) EDS map of carbon (blue) and sodium (green) superimposed on the SEM micrograph depicted in (**a**). (**c**,**d**) Individual elemental maps of carbon (**c**, blue) and oxygen (**d**, white). (**e**) Averaged spectrum of six independent single-spot EDS measurements. Scale bars, 5 μm (**a**–**d**).

**Figure 5:**
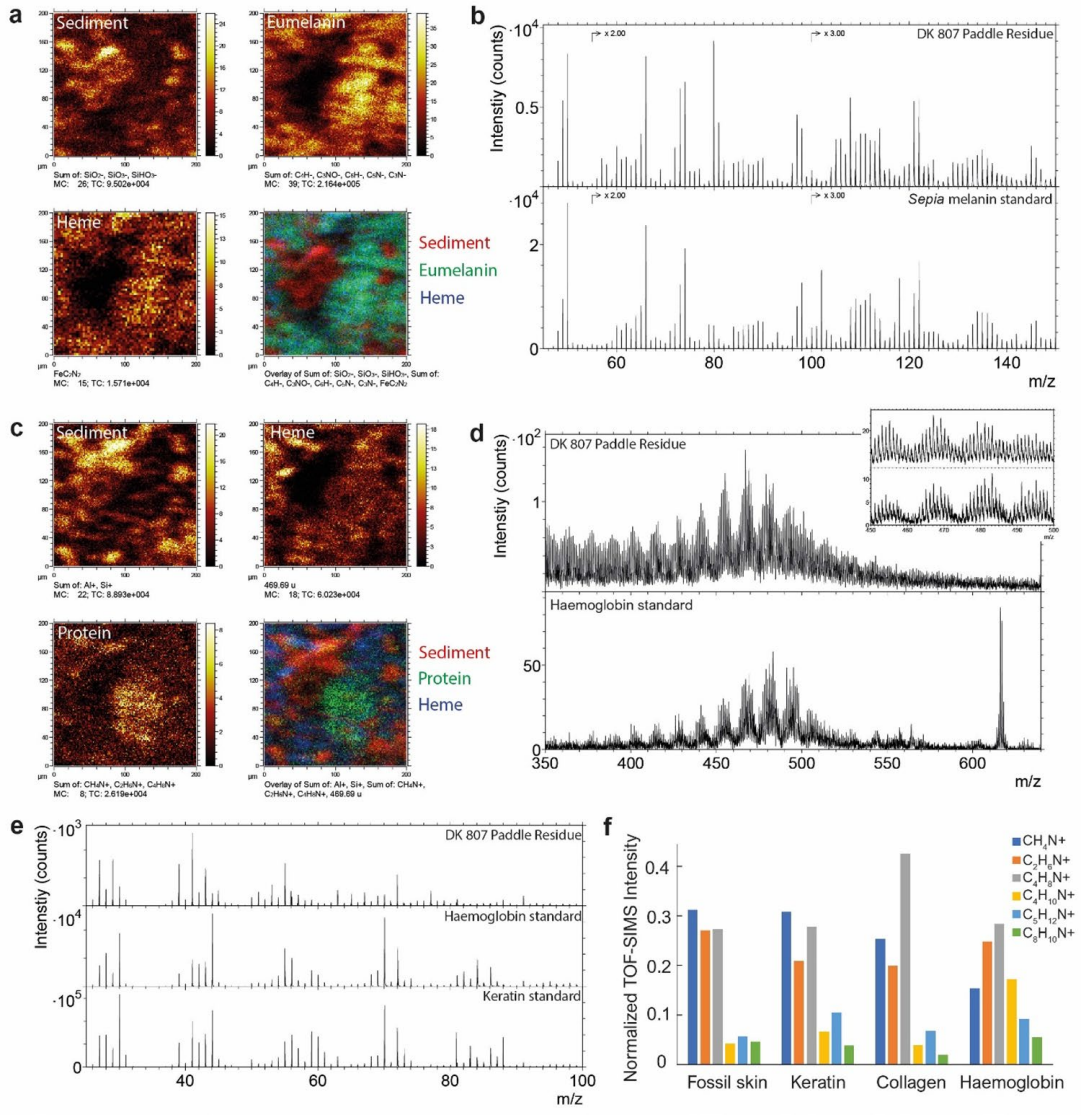
ToF-SIMS data obtained from the flipper residues in DK 807. (**a**) Negative-ion images showing the spatial distribution of ions representing sediment (SiO_2_^−^, SiO_3_^−^ and SiHO_3_^−^), eumelanin (C_4_H^−^, C_3_NO^−^, C_5_N^−^ and C_3_N^−^) and heme (FeC_2_N_2_^−^), respectively. The last panel is a three-colour overlay of the preceding figures, where the sediment is depicted in red, eumelanin in green and heme in blue. (**b**) Negative-ion spectrum acquired from an area of the fossil soft tissue with prominent eumelanin signal compared against a *Sepia* eumelanin standard spectrum. (**c**) Positive-ion images of the same area as depicted in (**a**), showing the spatial distribution of ions representing sediment (Al^+^, Si^+^), heme (*m/z* 435–502), and proteinaceous matter (CH_4_N^+^, C_2_H_6_N^+^ and C_4_H_8_N^+^). The final panel is an overlay image in which the sediment occurs in red, proteinaceous matter in green and heme in blue. (**d**) Positive-ion spectra of heme-associated ions from the fossil sample compared against a haemoglobin standard. Note the undulating pattern in both spectra representing Fe-porphyrin fragment ions characteristic of heme. (**e**) Positive-ion spectrum from a region of the fossil tissue with prominent signal from nitrogen-containing ions indicative of proteinaceous matter compared against spectra of haemoglobin and keratin standards. (**f**) Normalized signal intensities of characteristic peptide/protein fragment ions in DK 807 together with relevant protein standards.

**Figure 6:**
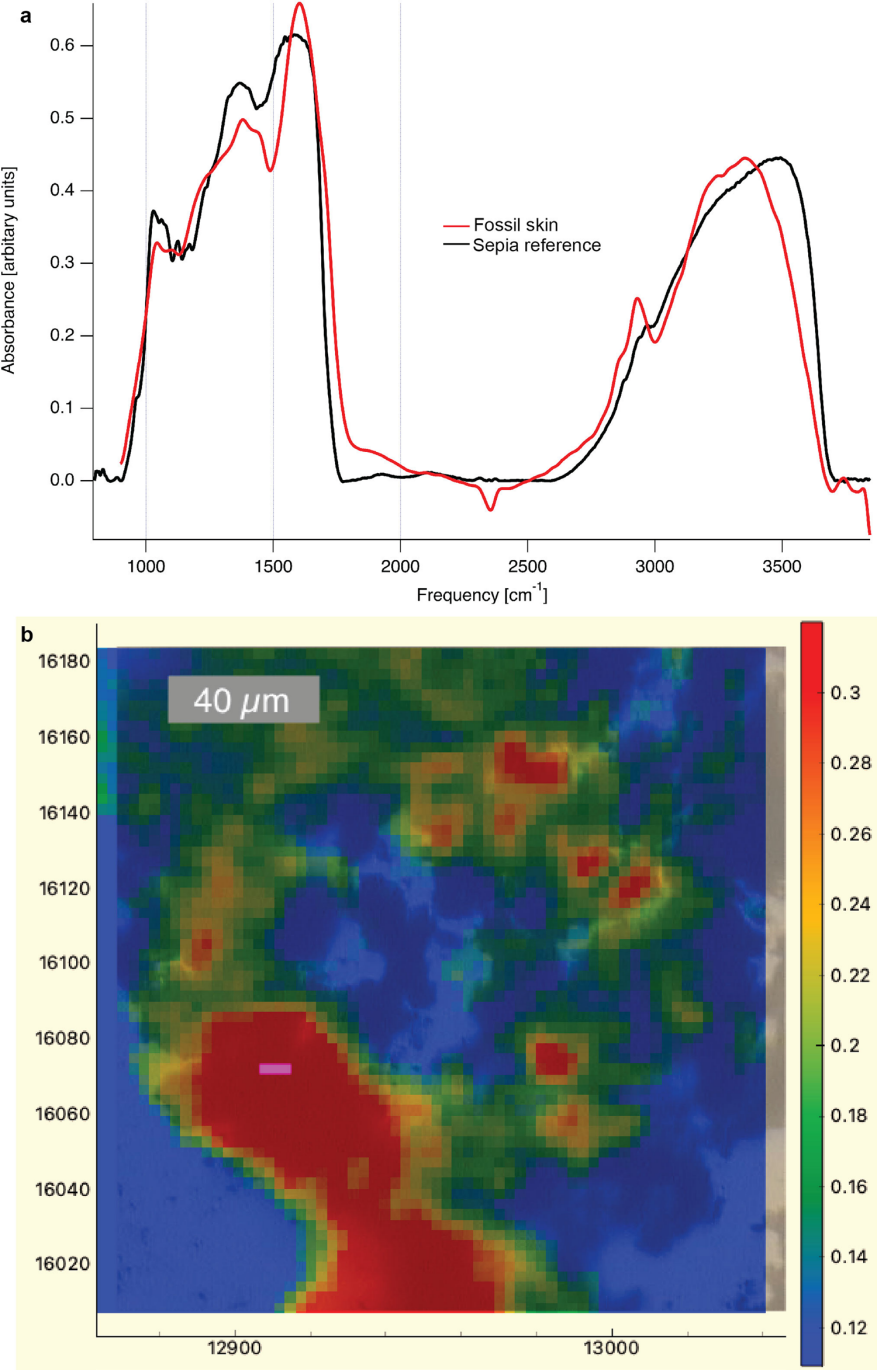
Infrared and optical images of a demineralized carbonaceous flipper residue from DK 807. (**a**) IR absorbance spectra from the residue together with a *Sepia* eumelanin reference. Three spectra, representing in total 192 scans (3 × 64) and originating from the purple region (3 × 1 pixels) in image (**b**) are averaged to improve the signal-to-noise ratio. Notice close similarity in broad-band absorbance in the 900–1800 and 2700–3700 cm^−1^ regions between the fossil and *Sepia* eumelanin standard. (**b**) Spatial distribution of the IR data superimposed onto an optical image (dark blue) of the demineralized flipper sample. The intensities of the IR image originate from the 1730 cm^−1^ absorbance band. A binomial (gaussian) smoothing was applied to suppress contributions from the water background^81^.

Energy dispersive X-ray microanalysis (EDS) of demineralized samples (Fig. 4a,b) showed a predominance of carbon (Fig. 4a,e) in the soft-tissue remains, with minor amounts of oxygen (Fig. 4d) and sodium (Fig. 4b,e). Subsequent molecular analysis by ToF-SIMS unveiled a suite of organics, including heme (Fig. 5a,c,d), residual eumelanin (Fig. 5a–c) and proteinaceous matter (Fig. 5c,e), as well as polyaliphatic and polyaromatic compounds. Molecular identification was possible by detailed analysis of the fossil ToF-SIMS spectra and comparisons with reference spectra, as previously demonstrated for various fossil soft tissues^19,28–33^. Negative-ion spectra indicated the presence of eumelanin in both scute (Supplementary Fig. S2) and flipper samples (Fig. 5b) as a set of fragment ions in the *m/z* 45–175 range with distinct relative intensity distributions^19,28–31^. This finding was corroborated by infrared microspectroscopy as broad-band absorbance in the 900–1800 and 2700–3700 cm^−1^ ranges (Fig. 6), which typically characterise this light-absorbing biochrome^19,28,34,35^.

ToF-SIMS analysis further identified heme via an undulating peak pattern between *m/z* 400 and 520 in positive-ion mode (Fig. 5d), as well as through specific iron- and nitrogen-containing fragment ions at, e.g., *m/z* 107.95 (Fe(CN)_2_^−^) in negative-ion mode^19,36^. Moreover, previously recognized peptide/protein fragment ions^19,32^, including CH_4_N^+^ (*m/z* 30.034), C_2_H_6_N^+^ (*m/z* 44.049), C_4_H_8_N^+^ (*m/z* 70.068), C_4_H_10_N^+^ (*m/z* 72.080), C_5_H_12_N^+^ (*m/z* 86.096) and C_8_H_10_N^+^ (*m/z* 120.072), were detected in positive-ion spectra from the paddle (Fig. 5e). Comparisons of the relative intensity distribution of these ions and those in spectra obtained from representative samples of haemoglobin, type I collagen and α-keratin revealed some similarities with keratin and collagen (Fig. 5f). However, this superficial resemblance should only be considered as indicative of proteinaceous matter, not as any specific protein per se, because these cannot be conclusively identified by ToF-SIMS alone^37,38^.

**Figure 7:**
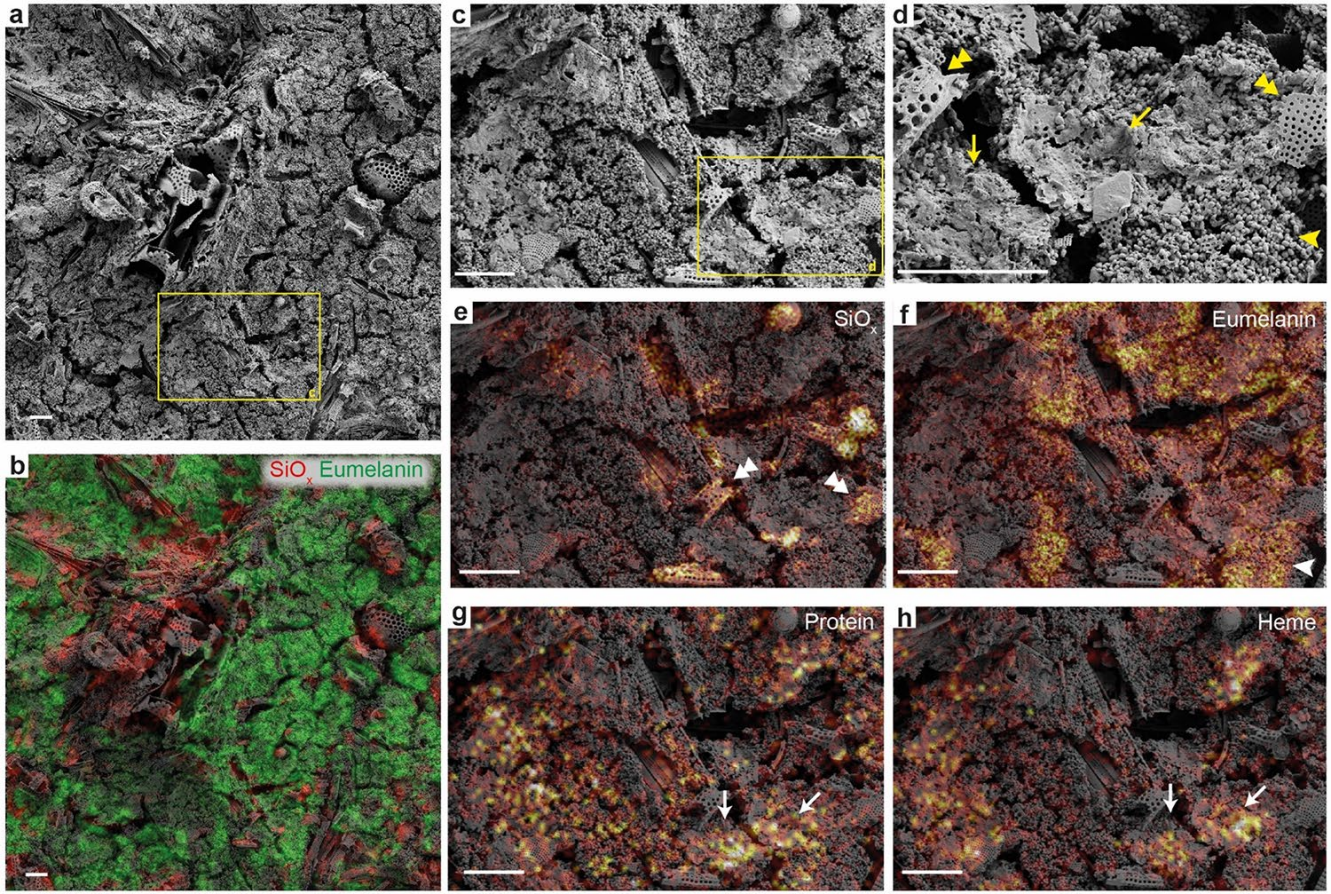
ToF-SIMS and SEM images of organic residues in demineralized samples obtained from the flipper residue in DK 807. (**a**) SEM micrograph of melanosomes and sheet-like matter overlying diatom frustules (the latter from the surrounding sediment). (**b**) Overlay of the SEM micrograph in (**a**) together with negative-ion images representing eumelanin in green (C_4_H^−^, C_3_N^−^, C_3_NO^−^, C_6_H^−^ and C_5_N^−^) and sediment in red (SiO_2_^−^, SiO_3_^−^ and SiHO_3_^−^). (**c**) Higher magnification SEM micrographs of the area denoted by a yellow box in (**a**). (**d**) Higher magnification SEM micrograph of the area indicated by a yellow box in (**c**). (**e**–**h**) Overlays of the SEM micrograph in (**c**) with individual ToF-SIMS ion images representing (**e**), silica (SiO_2_^−^, SiO_3_^−^ and SiHO_3_^−^); (**f**) eumelanin (C_4_H^−^, C_3_N^−^, C_3_NO^−^, C_6_H^−^ and C_5_N^−^); (**g**) proteinaceous matter (CH_4_N^+^, C_2_H_6_N^+^ and C_4_H_8_N^+^), and (**h**) heme (*m/z* 435–470, positive ions). Notice precise spatial correlations of the different molecular components with specific object types as indicated by double arrowheads for diatoms, arrowheads for melanosomes, and arrows for the organic matrix. Scale bars, 10 μm (**a**–**h**).

Positive-ion spectra further evinced the presence of both polyaliphatics (C_x_H_y_^+^, X < Y)^19^ and polyaromatics (C_x_H_y_^+^, X > Y)^19^ in tissue samples from the scute and paddle. Notably, the spatial distributions of eumelanin, heme and proteinaceous matter were associated with different microstructural features (Fig. 7). Overlays of ToF-SIMS ion maps and FEG-SEM micrographs demonstrated co-localization between eumelanin and the microbodies described above (Fig. 7d,f, arrowheads), whereas heme and peptides/protein fragments co-occurred with the adjacent fibrous/spongious matrix (Fig. 7d,g,h, arrows). The surrounding sedimentary matrix yielded primarily inorganic elements, including silicon oxides (SiO_X_^−^) associated with diatom frustules (Fig. 7d,e, double arrowheads).

## Discussion

**Figure 8:**
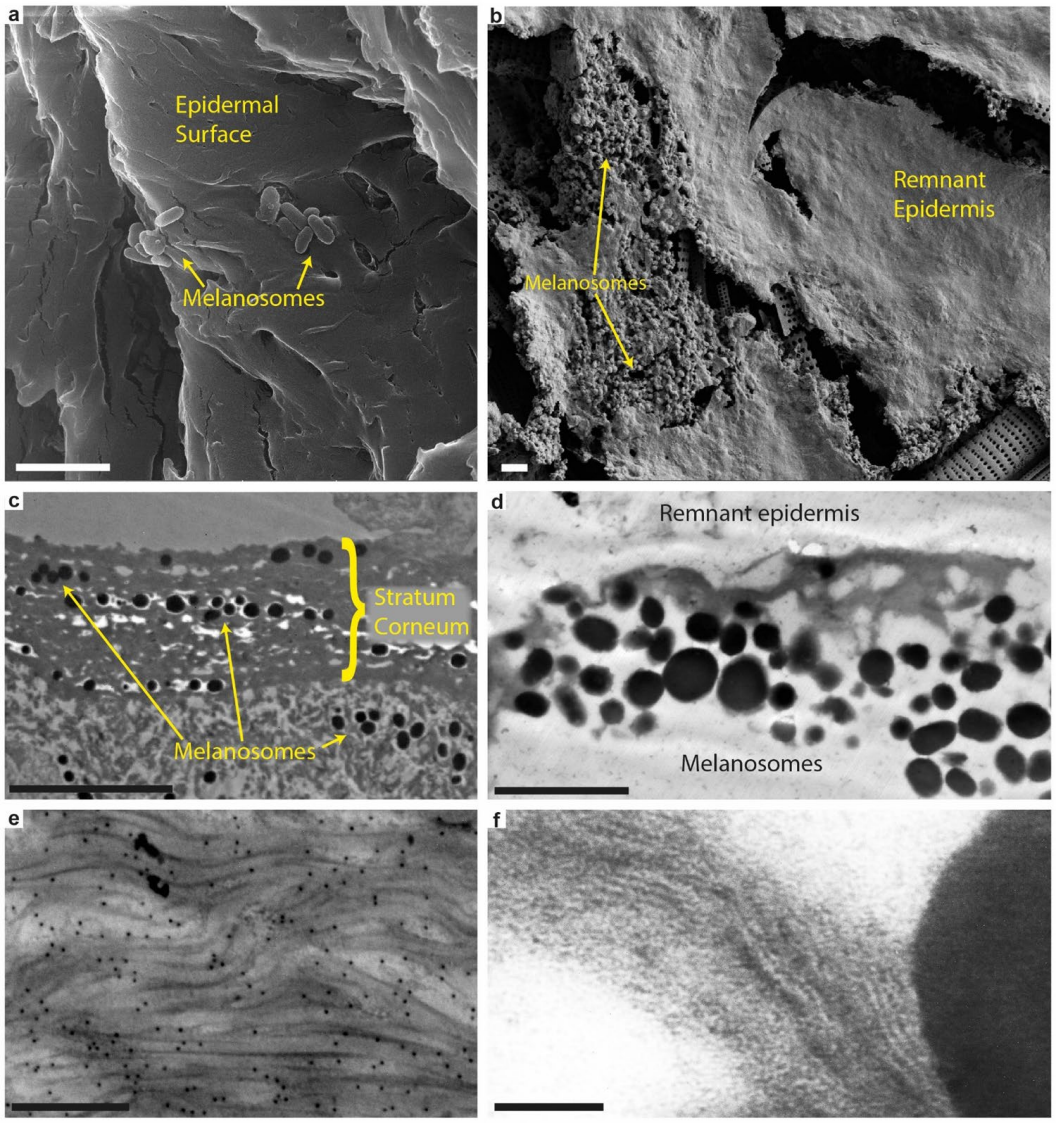
Side-by-side comparison of electron micrographs between modern sea turtle epidermis (**a**,**c**,**e**) and DK 807 flipper tissues (**b**,**d**,**f**). (**a**) SEM micrograph of the skin covering in a hatchling loggerhead sea turtle, *Caretta caretta*, showing melanosomes partially embedded in layered cornified proteins within the epidermis. (**b**) FEG-SEM micrograph of DK 807 flipper residue with swaths of sheet-like organic matter that partially covers remnant melanosomes. (**c**) TEM micrograph of carapace epidermis from a hatchling leatherback turtle, *Dermochelys coriacea*. (**d**) TEM micrograph of DK 807 flipper skin residue. (**e**) TEM micrograph of the carapace epidermis of a juvenile loggerhead sea turtle, *Caretta caretta*, with corneous protein filaments. (**f**) Close-up TEM micrograph of filamentous matter in the fossil flipper residue. Scale bars, 2 μm (**a**,**b**,**d**), 10 μm (**c**), 500 nm (**e**), 100 nm (**f**).

Residual eumelanin is a major geochemical component of the soft parts in DK 807, and invariably associated with micrometre-sized bodies (Fig. 7b,d,f) that confidently can be identified as remnant melanosomes^30^. These occur embedded in a fibrous to sheet-like matrix that morphologically compares favourably with partially degraded sea turtle integument (compare side-by-side images of extant (Fig. 8a,c,e) and fossil (Fig. 8b,d,f) epidermis). Moreover, the densely stacked laminae (Fig. 8f) are structurally consistent with keratin filaments present in the stratum corneum and stratum intermedium (the outer and middle layer of the epidermis, respectively) of extant turtle skin (Fig. 8c,e; see also Ref.^39^), where they contribute to its cornified exterior. Although some clay minerals (e.g., illite) can have a superficially similar layered appearance when visualized under TEM^40,41^, the organic composition of the matrix (Figs. 4, 7g,h) and our use of hydrofluoric acid (which dissolves silicates^42^) when liberating the soft-tissue remains from the surrounding host rock (see “Methods”) preclude an inorganic origin. Consequently, we interpret the carbonaceous flipper residue as fossilized skin in which the keratinous components of the epidermis have undergone incomplete diagenetic alteration into polymeric substances (hence our detection of both aromatics^43^ and putatively endogenous protein fragment ions^19,32^). The amassed melanosomes likely originate from both epidermal and dermal melanophores; however, while epidermal melanosomes seemingly form the bulk of the dark, sheet-like halo around the bones in the right flipper of DK 807, dermal melanosomes predominantly occur in patches of dense blackish matter in some areas (Fig. 2e,f). During the biostratinomy of DK 807, blood and/or myoglobin breakdown products (heme) from dermal and/or sub-dermal tissues presumably leaked into the decomposing epidermis, thereby contributing to the structural stability of the chemically transformed keratin filaments^19,44–47^.

**Figure 9:**
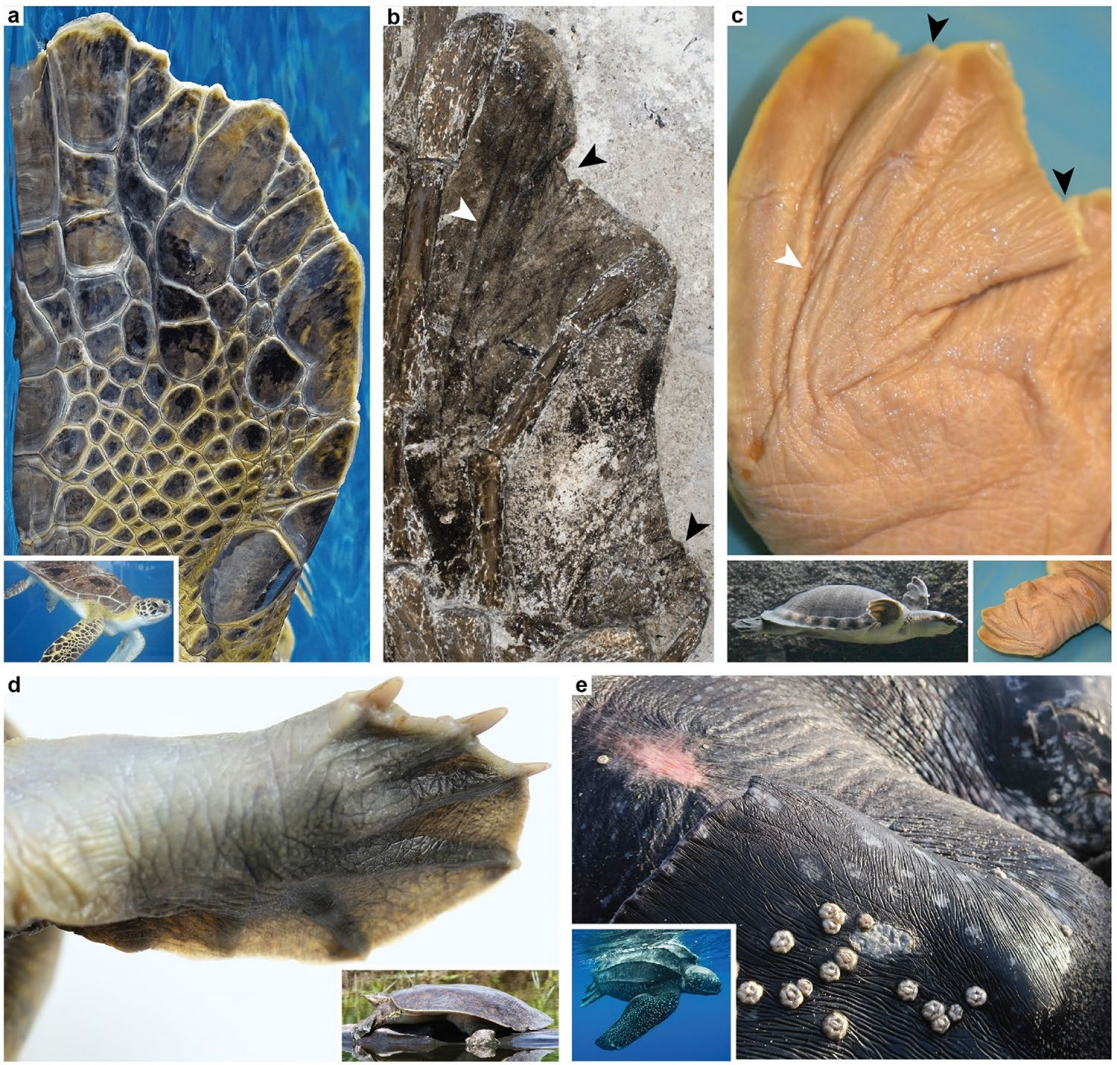
Comparisons of integumental patterns in various turtle paddles and flippers. (**a**) Rear flipper in a green sea turtle, *Chelonia mydas*, with scale patterns typical of extant cheloniid turtles. Inset, an adult green sea turtle. (**b**) Close-up image of the right rear flipper in DK 807 (compare with **c**; note arrowheads of corresponding colour indicating similarities in skin folds between the fossil and extant turtle flippers). (**c**) Fore-paddle in a modern pig-nosed turtle, *Carettochelys insculpta* (photo: Herpetology/Senckenberg Dresden). Note soft, scaleless skin with multiple folds. Inset, a pig-nosed turtle. (**d**) Scaleless manus of an Asiatic softshell turtle, *Amyda cartilaginea* (photo: dwi putra stoc/shutterstock.com). Inset, spiny softshell turtle, *Apalone spinifera* (photo: Ken Sturm/U.S. Fish and Wildlife Services/Public Domain). (**e**) Front flipper in an adult leatherback turtle, *Dermochelys coriacea* (photo: William Farah/shutterstock.com). Note leathery skin without scales. Inset, a swimming leatherback turtle (photo: NOAA Fisheries/Public Domain).

The webbed outline of the preserved flipper in DK 807 closely resembles rear paddles in living sea turtles (compare Figs. 2a and 9a). However, there are no visible remnant scales in this soft-tissue residue (Figs. 1, 2a, 8b), despite recent classification of DK 807 as a Pan-Cheloniidae^21^. This is surprising given that DK 807 has scutes on its shell, and that other previously documented hard-shelled sea turtles from the Eocene, including *Eochelone*, have deep sulci grooves on their skulls to suggest the presence also of facial scutes (e.g., Refs.^25,48^). Thus, it is reasonable to assume that these turtles also had scales covering their flippers (in similarity with extant hard-shelled sea turtles). However, from detailed comparisons with the modern pig-nosed turtle, *Carettochelys insculpta* (Fig. 9c), soft-shelled turtles (e.g., *Amyda cartilaginea* and *Apalone spinifera*; Fig. 9d) and leatherback turtle, *Dermochelys coriacea* (Fig. 9e), the phalangeal halo in DK 807 appears to comprise soft, partially folded skin (compare Fig. 9b and c) that has become secondary flattened by lithostatic pressure following decay and loss of most internal structures.

An alternative interpretation for the apparent absence of scales in DK 807 is a preservational bias favouring deeper integumental layers. In this scenario, the scaly outer epidermis was lost already during the initial stages of decay of the cadaver (if it, for instance, was floating around for some time before settling at the seafloor), leaving behind a dermis/sub-dermis that had lost much of its original structural integrity. However, even though we cannot completely rule out a biostratinomic cause for the apparent lack of scales in DK 807, this hypothesis is hard to reconcile with the preserved carapace scutes—which essentially are large and heavily cornified scales—and the results of our structural and molecular analyses (demonstrating that epidermal filamentous matter constitutes a substantial part of the soft tissues in the hind limbs). Both taphonomic experiments^49–51^ and field observations of reptile carcasses^10,52^ have shown that the epidermis, especially parts covering the limbs^10^, normally outlasts other non-biomineralized tissue types, particularly if entombed within sediments. Furthermore, when originally present, scales (and other keratinous epidermal structures, such as feathers) also are preferentially preserved in fossil vertebrates^38,47^. In part, this is likely a consequence of their chemical composition—comprising mostly tough keratinous components and recalcitrant melanic pigments^19,38^—but also because of the close spatial proximity between these integumental appendages and the surrounding host rock, contributing, e.g., to tissue fixation and protection from hydrolytic damage (see Ref.^19^ and references therein).

It is further noteworthy that the colours of the flipper substrate in DK 807 do not match pigmentation patterns typically seen in the scaly skin of extant sea turtles (Fig. 9a). This may be an artefact of preservation; however, in living reptiles with non-overlapping scales (such as sea turtles), darker pigmentations often occur underneath the scales themselves, whereas the thinner and more flexible hinge areas are lighter pigmented (Fig. 9a)^2,53^. The reason for this marked divergence is due to differences in melanosome density; melanosomes normally concentrate in the basal regions of scales, whereas the hinges generally lack dense aggregations of these cellular organelles^2,53^. Likewise, original pigmentation (melanin) patterns can explain why scales often can be seen in fossil reptiles with organically preserved skin^29,54,55^; an observation that is further compounded by the preserved scutes in DK 807. Importantly, complete decomposition of the integument, save for the toxic melanosomes^56^, would presumably result in a semi-continuous, more-or-less featureless mat of remnant pigment organelles without apparent folds and lineations, as seen in some heavily degraded organic soft-tissue fossils^57^.

Among living turtles, forms that have reduced or completely lost their scales and scutes are found in the aquatic Trionychidae (soft-shelled turtles), Carettochelyidae (pig-nosed turtle) and Dermochelyidae (leatherback turtle)^9–13^. Although integumental adaptations associated with life in the water remain incompletely understood^58^, it is generally believed that scale miniaturization or loss contribute to frictional drag reduction^54,58–60^, enhanced flow separation^58^ and/or increased limb/torso flexibility^59^, which in turn facilitates efficient swimming^9,54,58–60^ (but see also Ref.^20^ for a different interpretation). Hence, scale reduction, or even a complete loss of horny epidermal appendages, has occurred independently in multiple distantly related secondarily aquatic reptile lineages, including the Mesozoic ichthyosaurs^59,61,62^, thalattosuchians^63^ and mosasaurs^54,60^.

Interestingly, though, none of the six extant cheloniid species has embarked on this evolutionary path, despite being almost exclusively marine. It is possible that the polygonal scales seen in these turtles serves to stiffen the flippers during locomotion, as has been proposed for another lineage of aquatic testudines, namely the Jurassic Thalassochelydia^20^. However, alternative explanations for the heavy scalation in extant hard-shelled sea turtles may also exist. For example, modern cheloniids frequently visit reef environments^64^, and some species, e.g., the hawksbill sea turtle (*Eretmochelys imbricata*) and green sea turtle (*Chelonia mydas*), are known to subsist on food items (e.g., sponges and algae) that are common in such habitats^64^. Thus, in similarity with certain sea snakes (e.g., *Aipysurus*)^65,66^, the retention of prominent scales and scutes in these turtles could be a consequence of abrasive hazards imposed by corals and other sharp objects in their nearshore feeding grounds^64,66^. By contrast, the leatherback turtle is rarely observed in reef environments^67^, but instead typically inhabits pelagic settings^10^, occasionally even venturing into deep^68^ and cool^69^ waters in pursuit of prey. A heavy scalation could also be attributed to needs for protection from large-sized predators, such as sharks^70^. Hard-shelled sea turtles mostly forage on rather immobile prey^10^, and are slow-moving relative to the more active dermochelyids^71,72^.

With its ostensible mosaic of scaleless (lower) limbs and a scute-covered bony carapace, DK 807 appears to deviate in epidermal morphology from both extant cheloniids and dermochelyids. Assuming that the ancestral condition for testudines included distal extremities covered in scales^2,5,7,17^, it is tempting to view this partial scale-loss as an adaptation towards an obligate marine existence. Species of *Eochelone* are thought to have populated coastal areas with infrequent excursions into pelagic environs^25^ (but see also Ref.^73^), an inference in agreement with the moderately aquatically-adapted bony support of the pelvis and extremities in DK 807^21^. Notably, these skeletal units share characteristics with *Toxochelys*^74,75^, a Cretaceous stem chelonioid that is considered to be transitional between coastal and fully pelagic forms^76^. Similarities in skeletal anatomy are found also with the webbed feet of extant soft-shelled and pig-nosed turtles, to suggest the presence of relatively flexible rear paddles in DK 807 with digits that could move independent of one another^21^, thereby enabling shape adjustments during locomotion^77,78^. As noted elsewhere^74^, early cheloniids (including *Eochelone*) presumably used their hind limbs to a greater extent when swimming than do their modern counterparts (in which the rear paddles chiefly serve as rudders and balance organs). It has even been hypothesized^75^ that these turtles could have alternated between typical fore flipper propulsion during cruising (otherwise seen in extant sea turtles)^74,75^ and thrust generated via synchronized lateral strokes by both the fore- and hind limbs (in similarity with modern freshwater turtles)^75,77^.

Despite a general agreement that the invasion of the marine realm by the ancestral pan-chelonioids occurred only once in the Cretaceous^16^, it has been suggested that certain adaptations, such as a reduced bony shell and specific modifications to the hind limbs, could have evolved independently in different sea turtle clades^74^. This has led some authors^74^ to propose the presence of a hitherto unidentified ghost lineage of coastal turtles that underwent multiple radiations into fully marine forms following the major sea turtle extinctions at the end of the Cretaceous and during the latest Eocene–earliest Oligocene interval^74,79^. Palaeocene and Eocene pan-cheloniids represent one such radiation, and their combination of primitive and derived skeletal features^21,74^ indicates progressive adaptations toward a facultative open-water life. The mosaic of integumental characteristics documented here in DK 807 likewise could constitute modifications that arose independently in this turtle lineage as a response to increased aquatic habits. However, because DK 807 represents a single (and additionally incomplete) individual, and because taphonomic influence cannot be ruled out, any broader palaeobiological implications of the seemingly scaleless skin in this fossil turtle remain hypothetical and thus need to be validated by additional soft-tissue specimens of similar age.

## Methods

### Contamination prevention

Great care was taken to prevent contamination at all stages of our analyses. For instance, modern and fossil samples were handled in segregated laboratory spaces using instruments designated specifically for each workroom. Medical-grade nitrile gloves, face masks and gowns were additionally worn when handling the fossil materials. Moreover, all working surfaces were covered with fresh aluminum foil, and tools (e.g. forceps, pipettes, and scalpels) were rinsed multiple times with 96% ethanol and Milli-Q water prior to manipulation of the fossil matter.

### Fossil materials

The fossil samples used in our analyses originate from counterslab pieces of DK 807 that were collected using a pneumatic scribe during the preparation of the fossil. These were not treated with any consolidant or adhesive; instead, they were immediately wrapped in aluminum foil pending our structural and/or molecular analyses.

Fossil samples were demineralized via treatment with either 0.5 M ethylenediaminetetraacetic acid (EDTA, pH 8.0; purchased from PanReac ApplicChem) or 40% hydrofluoric acid (HF; purchased from VWR). Materials treated with EDTA were immersed in glass jars and left to react for two weeks with daily buffer changes. Samples treated with HF were submerged overnight in a HF-compatible plastic tray (6-well Tissue Culture Plates purchased from VWR). The following day, the HF was replaced with EDTA and allowed to sit over the course of three days. Milli-Q water was used to wash all samples 9 times after demineralization.

### Comparative materials

Tissue samples used for comparisons included: (1) epidermis from the carapace of a hatchling *Caretta caretta* (KPC16030906; fixed in 10% formaldehyde) found dead in the Kyoto Prefecture of Japan in 2010; and (2) skin from a neonate *Dermochelys coriacea* (ZMUC R2106; stored in 70% ethanol) donated as a gift from ‘Danmarks Akvarium’ (former Danish Aquarium) to the Zoological Museum, Natural History Museum of Denmark.

### Photography and light microscopy

Specimen pictures were taken with a Nikon D3500 equipped with a standard 18–55 mm zoom lens mounted on top of a tripod. A polarized light source was used together with a camera-attached circular polarized filter to increase contrast between fossil soft-tissue and matrix. Close up photos of soft tissues were taken by using a 60-mm macro lens. Untreated and demineralized samples smaller than 2 cm were examined using an Olympus SZX16 microscope with an Olympus SC30 digital camera.

### Scanning electron microscopy

Initial imaging was done in a Tescan Mira3 High Resolution Schottky FEG-SEM at the Department of Geology, Lund University, Sweden. Fossil tissues (both untreated and demineralized samples) and modern reference materials were mounted on metal stubs with carbon tape, and coated with a 12-nm-thick mixture of platinum and palladium. Working distance was 3–15 mm at 15–30 keV using both in-beam and standard secondary electron detectors. Following ToF-SIMS analysis (see below), additional imaging was conducted in a Zeiss Supra 40VP FEG-SEM using 2 keV electron energy, a working distance of 3–5 mm, and an Everhart–Thornley secondary electron detector.

### Transmission electron microscopy

Tissues from extant sea turtles were immersed in 2.5% glutaraldehyde overnight at 4 °C and then rinsed with 0.25 M sucrose in 0.1 M phosphate buffer, followed by immersion in 1% osmium tetroxide for two hours at room temperature (for lipids). These fixation steps were excluded for the fossil samples as the recalcitrant organic material typically withstands the electron beam. After overnight fixation, the samples were dehydrated and then stepwise embedded in an epoxy resin/ethanol mixture until only pure resin remained. They were polymerized at room temperature for 72 h and then again at 60 °C for 48 h. Thin sections were produced with a Leica EM UC7 ultramicrotome using a glass knife for semi-thin sections and a diamond knife for ultrathin sections. Semi-thin sections (200-nm-thick) were made for quality control and further analysis under a light microscope. Ultrathin sections (50-nm-thick) were prepared for TEM and mounted onto pioloform-coated copper grids. Both modern and demineralized fossil tissue samples were stained with uranyl acetate and lead citrate to increase contrast. TEM analyses were conducted using a JEOL JEM-1400 PLUS instrument at 80 and 120 kV at the Department of Biology, Lund University, Sweden. Images were taken with a bottom-mounted Matataki CMOS camera.

### Time-of-flight secondary ion mass spectrometry

ToF-SIMS is a chemical analysis method that identifies molecular species on sample surfaces by irradiating them with a focused beam of high energy (primary) ions and acquiring mass spectra of the ejected (secondary) ions^80^. By scanning the primary ion beam over a selected analysis area and recording mass spectra from each pixel (typically 256 × 256 or 512 × 512 pixels) ion images are generated that show the signal intensity distribution of specific secondary ions (representing specific molecular species) on the analysis area; alternatively, mass spectra from selected regions of interest are collected for molecular characterization of specific structures/regions on the sample surface^38,80^.

Samples from the right flipper and a scute in DK 807 were fixed on silicon substrates using double-sided tape and analyzed without further treatment in a TOFSIMS IV instrument (IONTOF GmbH) using 25 keV Bi_3_^+^ primary ions and low energy electron flooding for charge compensation. Positive and negative ion data were acquired under static SIMS conditions; that is, at primary ion dose densities for which the sample surface can be considered unaffected by damage caused by the primary ions (below the so called static limit; i.e., 10^12^–10^13^ ions/cm^2^), with the instrument settings optimized for either high mass resolution (m/Δm ~ 5000, spatial resolution ~ 3–4 μm,) or high image resolution (m/Δm ~ 300, spatial resolution ~ 0.2–0.5 μm). High mass resolution data were acquired with a pulsed primary ion current of 0.10 pA, at raster areas of 200 × 200 µm^2^ (128 × 128 pixels) to 500 × 500 µm^2^ (256 × 256 pixels), and acquisition times up to 123 s, resulting in primary ion dose densities below 2 × 10^11^ ions/cm^2^, whereas high image resolution data were acquired with a pulsed primary ion current of 0.04 pA, at raster areas of 200 × 200 µm^2^ (512 × 512 pixels) and acquisition times up to 1311 s, resulting in primary ion dose densities below 10^12^ ions/cm^2^. Results were compared against spectra from various standards, including natural eumelanin from *Sepia officinalis*, α-keratin, haemoglobin, and type 1 collagen (all purchased from Sigma-Aldrich).

### Infrared microspectroscopy

Demineralized fossil samples were suspended in water, loaded onto sterile CaF_2_ infrared windows, and allowed to air dry overnight under a hood. They were then anaylzed at the Department of Biology, Lund University, using a Hyperion 3000 microscope combined with a Tensor 27 spectrometer equipped with a 64 × 64 pixel focal plane array (FPA) detector and a Globar light source. The microscope was operated in transmission mode at 4 cm^−1^ resolution and a × 15 objective was used. 64 individual scans were averaged.

### Ethics statement

No live animals were used in this study; instead, tissue sampling occurred only on dead museum specimens. Photographs derive either from stock images, museum specimens, or turtles on public display in aquarium tanks.

## Supplementary Information

Supplementary Information.

## Data Availability

The datasets generated during the study are available from the corresponding author upon reasonable request.
