# Supplementary material for: An ancestral hard-shelled sea turtle with a mosaic of soft skin and scutes
Source: Sci Rep. 2022 Dec 31;12:22655. doi: 10.1038/s41598-022-26941-1 (PMC9805447; doi:10.1038/s41598-022-26941-1)
Supplement: Supplementary file 1 — Supplementary Information. [file 41598_2022_26941_MOESM1_ESM.docx]

**Fossilized turtle soft tissues.** Turtle soft tissues are exceedingly rare in the fossil record. Reported occurrences are summarized in Table S1 below.

| Locality | Country | Age | Taxon (Specimen ID) | Notes | Reference |
| --- | --- | --- | --- | --- | --- |
| Stonesfield Slate | United Kingdom | Middle Jurassic (Bathonian) | Undetermined basal taxon (BMNH 37218, R247,39198, R5320) | Isolated scutes | 1 |
| Tiaojishan Formation | China | Late Jurassic (Oxfordian) | Unnamed turtle (PKUP V1059) | Patches of carbonaceous matter surrounding a disarticulated limb | 2 |
| Torleite Formation | Germany | Late Jurassic (Late Kimmeridgian) | Thalassochelyidian: *Thalassemys bruntrutana* (NKMB Watt 18/211, ) | Patches of either phosphatized or imprints of soft tissues on the forelimbs, hind limbs, skull, neck, and tail | 3 |
| Altmühltal Formation (Solnhofen Plattenkalk) | Germany | Late Jurassic (Early Tithonian) | Numerous thalassochelyidians: *Thalassemys* sp. (JME 3995), *Eurysternum wagleri* (BSPG 1969), *Palaeomedusa testa* (MB R 2894) | JME 3995 shows dermal remains around the pes with impressions of scales. BSPG 1969 displays impressions of scales in the area of one elbow. MB R 2894 has impressions of skin covering the neck | 3, 4, 5 |
| Crato Formation | Brazil | Early Cretaceous (Aptian) | Pleuodire: *Araipemys aturi* (SMNK-PAL 3979), an undetermined juvenile turtle (MB.R.3448) | SMNK-PAL 3979 preserves an outline of a foot with phosphatized muscle fibers. MB.R.3448 has soft tissues preserved in the neck, one limb and body | 6 |
| Nammoura site | Lebanon | Late Cretaceous (Cenomanian) | Protostegid: *Rhinochelys nammourensis* (ESC-2) | Extensive soft-tissue preservation in the paddles (both forelimbs and hind limbs) and tail | 7 |
| Clapp Creek | United States | Late Cretaceous | Bothremydid: *Taphrosphys sulcatus* (CMM-V-4524) | Impressions of scutes on a coprolite | 8 |
| Nacimiento Formation | United States | Paleocene | Baenid: *Neurankylus eximius* (LACM 127773) | Shell covered with pigmented scutes | 9 |
| Fur Formation | Denmark | Early Eocene | Cheloniid: *Tasbacka danica* (DK 567/MHM-K2), Dermodchelyid: *Eosphargis breineri* (FUM-N-1450) | DK 527/MHM-K2 shows extensive soft-tissue remains in the flippers, head and shell (complete with scutes). FUM-N-1450 displays carbonaceous tissue residues in the posterior part of the body | 10, 11,12 |
| Messel Pit | Germany | Middle Eocene | Geomydids: "*Ocadia*" (HLMD-Me 8051, 8877, 14749, 15033, 15565, 15577) | Several specimens with three-dimensionally preserved uteri | 13 |
| Valentine Formation | United States | Miocene | Emydid: *Chrysemys picta* (UNSM 76564) | Shell with purported pigment markings on the carapace | 14 |

Of the above listed specimens, DK 567^10^, the baenid (LACM 127773)^9^, Stonesfield Slate scutes^1^ and thalassochelydians (JME 3995 and BSPG 1960)^3,4^ from the Solnhofen Plattenkalk have all been noted for preserving scales. In DK 567, marginal scutes extend from the bony borders of the carapace^10^, whereas the baenid has epidermal scales that cover roughly 85% of the carapace, with many of these integumental appendages showing a reddish-brown colouration demarcated by black spots^9^. The thalassochelyidans show extensive impressions of scalations associated with the appendicular skeletons^3-5^, with JME 3995 in particular showing flat polygonal scales of varying sizes^3^. Despite being only distantly related to pan-cheloniids, these scales display a remarkable similarity to those of extant hard-shelled sea turtles, a trait that has been attributed to convergence following adaptations to life in marine environments^3^.

**Taphonomy of DK 807.** The fossilization of the epidermis in DK 807 likely was aided by a combination of preservational pathways. External geochemical factors, such as concretion formation and adhering clay particles, already have been discussed in a previous work^15^. However, the integument of DK 807 also had intrinsic factors that might have contributed to its long-term survival.

Decay experiments using extant vertebrate carcasses have shown the persistence of skin and its derivative structures (e.g., scales and feathers) even at advanced stages of decay^16,17^. In particular, it has been shown that in decaying turtles (as well as in other reptiles), the integument covering the head and abdominal cavity often is rapidly lost, causing the skeleton in these areas to be the first to become disarticulated^16,17^. Skin on the limbs, however, often remains intact long after internal organs and connective tissues are gone. Additionally, scutes – due to their thick keratinous corneous layer – are highly resistant to decay, although individual elements tend to peel off the shell^18^. These observations are consistent with the preserved soft-tissue structures in DK 807 (Figs. 1, S1a and 3a).

The skin is resilient to external physiochemical forces in part because of recalcitrant biomolecules, such as keratins and eumelanin^19,20^. The survival of melanins in the fossil record is well documented (see Ref. 19 and references therein), and additionally has been the subject of numerous taphonomical experiments^21–23^. This biochrome is a polymer, formed from highly polymerized cross-links with heterocyclic structures that are well suited for mitigating a variety of hazards, including ultraviolet light (see Ref. 23 and references therein). Keratins, on the other hand, are structural proteins that incorporate robust intra- and intermolecular crosslinks which are aided in part by cysteine sulphide bridges^20,25^. While the preservation of keratins in the fossil record remains a controversial topic^26,27^, decay experiments under a variety of conditions repeatedly have shown that these proteins have excellent preservation potential due to their hydrophobic nature and resistance to abrasive hazards^25,28^. Heme may also have played a role in the preservation of the inferred epidermal proteins in DK 807. Biogenic iron released from degrading haemoglobin and myoglobin could have infiltrated the epidermal tissues during early stages of decay and catalysed the formation of free radical oxidation reactions, which in turn could have promoted protein crosslinking^29,30^.

**Comparison between scute and flipper residues**

Analysis of the carbonaceous residues from both the flipper and scute revealed noticeable differences in the ultrastructural and biochemical content of the two tissue types. Macroscopically, samples obtained from the scute were darker in appearance than the flipper residue, and completely lacked lineations (compare Fig. 2b–d with Supplementary Fig. S1b). This can likely be explained by the abundance of melanosomes at the microscale in the scute tissues, with little or no fibrous to spongious matrix, as otherwise seen in the flipper residue (Supplementary Fig. S1c–e). What remains of the fibrous matrix in the scute reside occurs as short fibres that additionally appear to be heavily degraded when visualized under electron microscopy (Supplementary Fig. S1e).

Our biochemical investigation further revealed significantly less heme in the scute than in the flipper samples (Supplementary Fig. S3), and no detectable proteinaceous matter. These differences could be a consequence of heme concentration. The flipper skin might have been exposed to blood (haemoglobin) and muscle (myoglobin) degradation products leaking into the integument post-mortem. The scute, however, being neither as vascularized nor in close association with muscle tissues, would have been less exposed to heme in purge fluids.

**References**

1. Anquetin, J. and Claude, J. Reassessment of the oldest British turtle: *Protochelys* from the Middle Jurassic Stonesfield Slate of Stonesfield, Oxfordshire, UK. *Geodiversitas* **30**, 331–344 (2008).
2. Li Q., Clarke, J.A., Gao, K., Zhou, C., Meng, Q., Li, D., D’Alba, L. and Shawkey, M.D. Melanosome evolution indicates a key physiological shift within feathered dinosaur. *Nature* **507**, 350–353 (2014).
3. Joyce, W.G., Mäuser, M. and Evers, S.W. Two turtles with soft tissue preservation from the platy limestones of Germany provide evidence for marine flipper adaptations in Late Jurassic thalassochelydians. *PLoS One* **16**, e0252355 (2021).
4. Anquetin, J. and Joyce, W.G. A reassessment of the Late Jurassic turtle *Eurysternum waleri* (Eucryptorira, Eurysternidae). *Journal of Vertebrate Paleontology* **34**, 1317–1328 (2014).
5. Joyce, W.G. A new Late Jurassic turtle specimen and the taxonomy of *Palaeomedusa testa* and Eurysternum walgeri. *PaleoBios* **23**, 1–8 (2003).
6. Fielding, S., Martill, D.M. and Naish, D. Solnhofen-style soft-tissue preservation in a

new species of turtle from the Crato Formation (Early Cretaceous, Aptian) of north-east Brazil. *Palaeontology* **48**, 1301–1310 (2005).

1. Tong, H., Hirayama, R., Makhoul, E. and Escuillié, F. Rhinochelys (Chelonioidea:

Protostegidae) from the Late Cretaceous (Cenomanian) of Nammoura, Lebanon. *Atti della Società Italiana di Scienze Naturali e del Museo Civico di Storia Naturale in Milano* **147**, 113–138 (2006).

1. Godfrey, S.J., Weems, R.E. and Palmer, B. Turtle shell impression in a coprolite from South Carolina, USA. *Ichnos* **26**, 20-27 (2019).
2. Sullivan, R.M., Lucas, S.G., Hunt, A.P. and Fritts, T.H. Color pattern on the selmacryptodiran turtle *Neurankylus* from the Early Paleocene (Purercan) of the San Juan Basin, New Mexico. *Contributions to Science* **401**, 1–9 (1988).
3. Nielsen, E. Eocene turtles from Denmark. *Meddelelser fra Dansk Geologisk Forening*

**14**, 96–115 (1959).

1. Lindgren, J., Sjövall, P., Carney, R.M., Uvdal, P., Gren, J.A., Dyke, G., Pagh Schultz, B.,

Schwkey, M.D., Barnes, K.R. and Polcyn, M.J. Skin pigmentation provides evidence of convergent melanism in extinct marine reptiles. *Nature* **506**, 484–488 (2014).

1. Lindgren, J., Kuriyama, T., Madsen, H., Sjövall, P., Zheng, W., Uvdal, P., Engdahl, A.,

Moyer, A.E., Gren, J.A., Kamezaki, N., Ueno, S. and Schweitzer, M.H. Biochemistry and adaptive colouration of an exceptionally preserved juvenile fossil sea turtle. *Scientific Report* **7**, 13324 (2017).

1. Gaßner, T., Micklich, N., Kohring, R. and Gruber, G. Turtles (Testudines, Geoemydidae, “*Ocadia*” sp.) with three-dimensionally preserved remains of internal organs from the Middle Eocene of Grube Messel (Hessen, Germany). *Kaupia* **11**, 111–123 (2001).
2. Holman, J.A. and Sullivan, R.M. A small herptofauna from the type section of the Valentine Formation (Miocene: Barstovian), Cherry County, Nebraska. *Journal of Paleontology* **55**, 138–144 (1981).
3. De La Garza, R.G., Madsen, H., Eriksson, M.E. and Lindgren, J. A fossil sea turtle (Reptilia, Pan-Cheloniidae) with preserved soft tissues from the Eocene Fur Formation of Denmark. *Journal of Vertebrate Paleontology* **41**, e1938590 (2021).
4. Jackson, F.D., Varricchio, D.J., Jackson, R.A., Walde, A.D. and Bishop, G.A. Taphonomy of extant Desert Tortoise (*Gopherus agassizii*) and Loggerhead Sea Turtle (*Caretta caretta*) nesting sites: Implications for interpreting the fossil record. *Palaios* **30**, 207–223 (2015).
5. Richter, A. and Wuttke, M. Analysing the taphonomy of Mesozoic lizard aggregates from Uña (eastern Spain) by X-ray controlled decay experiments. *Palaeobiodiversity and Palaeoenvironments* **92**, 5–28 (2012).
6. Witherington, B.E. and Witherington, D. Our sea turtles: A practical guide for the Atlantic and Gulf, from Canada to Mexico. (Pineapple Press, Sarasota, Florida, USA, 2015)
7. Vinther, J. Reconstructing vertebrate paleocolor. *Annual Review of Earth and Planetary Sciences* **47**, 345–375 (2020).
8. Schweitzer MH. Soft tissue preservation in terrestrial Mesozoic vertebrates. *Annual Review of Earth and Planetary Sciences* **39**, 187–216 (2011).
9. McNamara M.E., Van Dongen, B.E., Lockyer, N.P., Bull, I.D. and Orr, P.J. Fossilization of melanosomes via sulfurization. *Palaeontology* **59**, 337–350 (2016).
10. Saitta, E.T., Kaye, T.G. and Vinther, J. Sediment-encased maturation: A novel method for simulating diagenesis in organic fossil preservation. *Palaeontology* **62**, 135–150 (2019).
11. Jarenmark, M., Sjövall, P., Ito, S., Wakamatsu, K. and Lindgren, J. Chemical evaluation of eumelanin by ToF-SIMS and Alkaline Peroxide HPLC analysis. *International Journal of Molecular Sciences* **22**, 161 (2021).
12. Negro, J.J., Finlayson, C. and Galván, I. Melanins in fossil animals: Is it possible to infer life history traits from the coloration of extinct species. *International Journal of Molecular Sciences* **19**, 230 (2018).
13. Schweitzer, M.H., Zheng, W., Moyer, A.E., Sjövall, P. and Lindgren, J. Preservation potential of keratin in deep time. *PLoS ONE* **13**, e0206569 (2018).
14. Saitta, E.T., Rogers, C., Brooker, R.A., Abbott, G.D., Kumer, S., O’Reilly, S.S., Donohoe, P., Dutta, S., Summons, R.E., and Vinther, J. Low fossilization potential of keratin protein revealed by experimental taphonomy. *Palaeontology* **60**, 547–556 (2017).
15. Saitta, E.T., Rogers, C.S., Brooker, R.A., and Vinther, J. Experimental taphonomy of keratin: A structural analysis of early taphonomic changes. *Palaios* **32**, 647–657 (2017).
16. Slater, T.S., McNamara, M.E., Orr, P.J., Foley, T.B., Ito, S. and Wakamatsu, K. Taphonomic experiments resolve controls on the preservation of melanosomes and keratinous tissues in feathers. *Palaeontology* **63**, 103–115 (2019).
17. Schweitzer, M.H., Zheng, W., Cleland, T.P., Goodwin, M.B., Boatman, E., Theil, E., Marcus, M.A. and Fakra, S.C. A role for iron and oxygen chemistry in preserving soft tissues, cells and molecules from deep time. *Proceedings of the Royal Society B* **281**, 20132741 (2014).
18. Boatman, E.M, Goodwin, M.B., Holman, H.N., Fakra, S., Zheng, W., Gronsky, R. and Schweitzer, M.H. Mechanisms of soft tissue and protein preservation in *Tyrannosaurs rex*. *Scientific Reports* **9**, 15678 (2019).

**Supplementary figure**
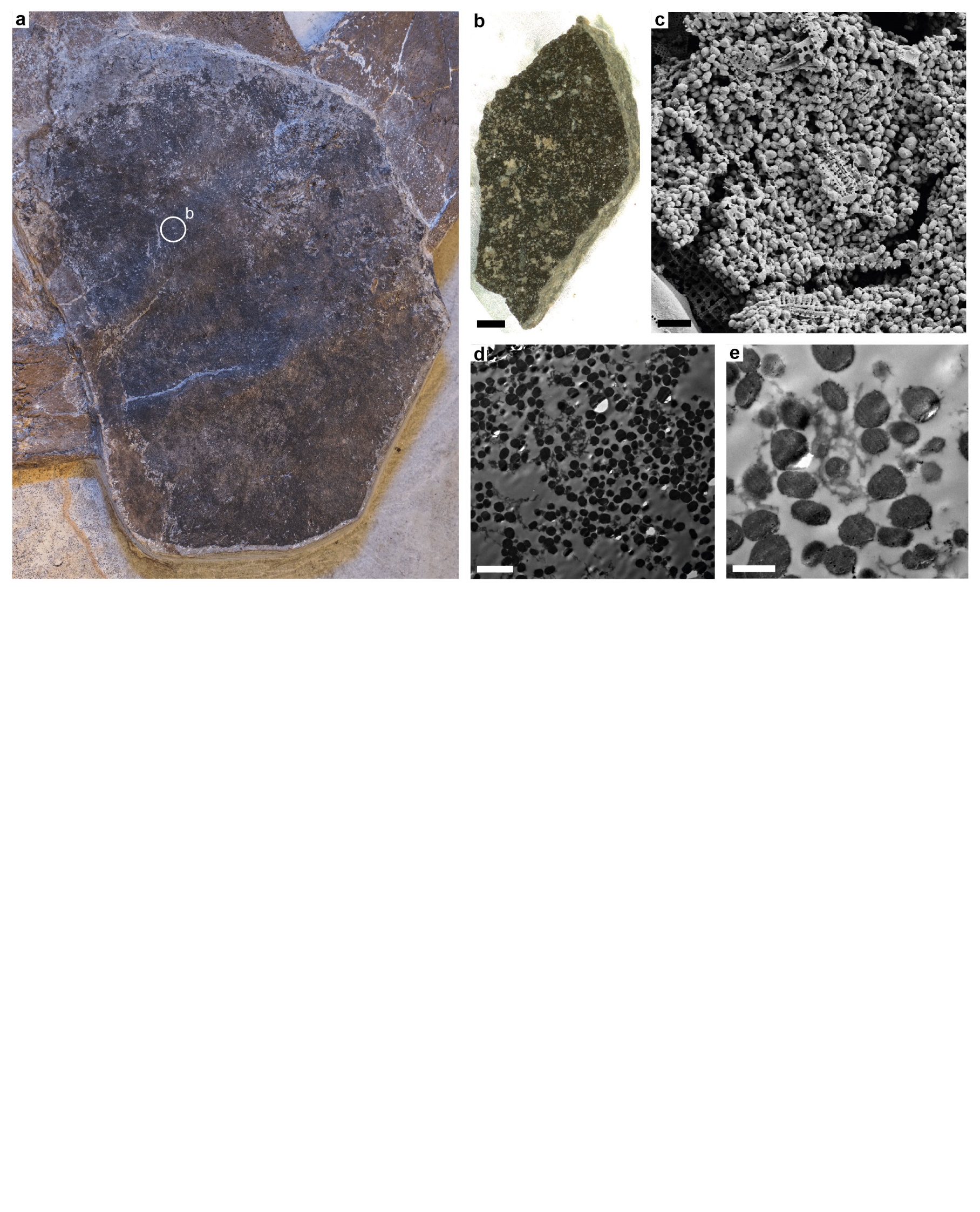


Figure S1. Macro- and ultrastructure of scute soft tissues. **a**, Photograph of scute residue under normal light. Sampled area is indicated by the white circle. **b**, Light-micrograph of the sampled scute residue. **c**, SEM micrograph of microbodies in the scute residue. **d**, TEM micrograph of the microbodies. **e**, TEM micrograph of the microbodies and associated fibrous matrix. Note the frayed appearance of the fibre-like structures located in between the microbodies. Scale bars, 500 μm (**b**), 2 μm (**c**, **d**), 500 nm (**e**).


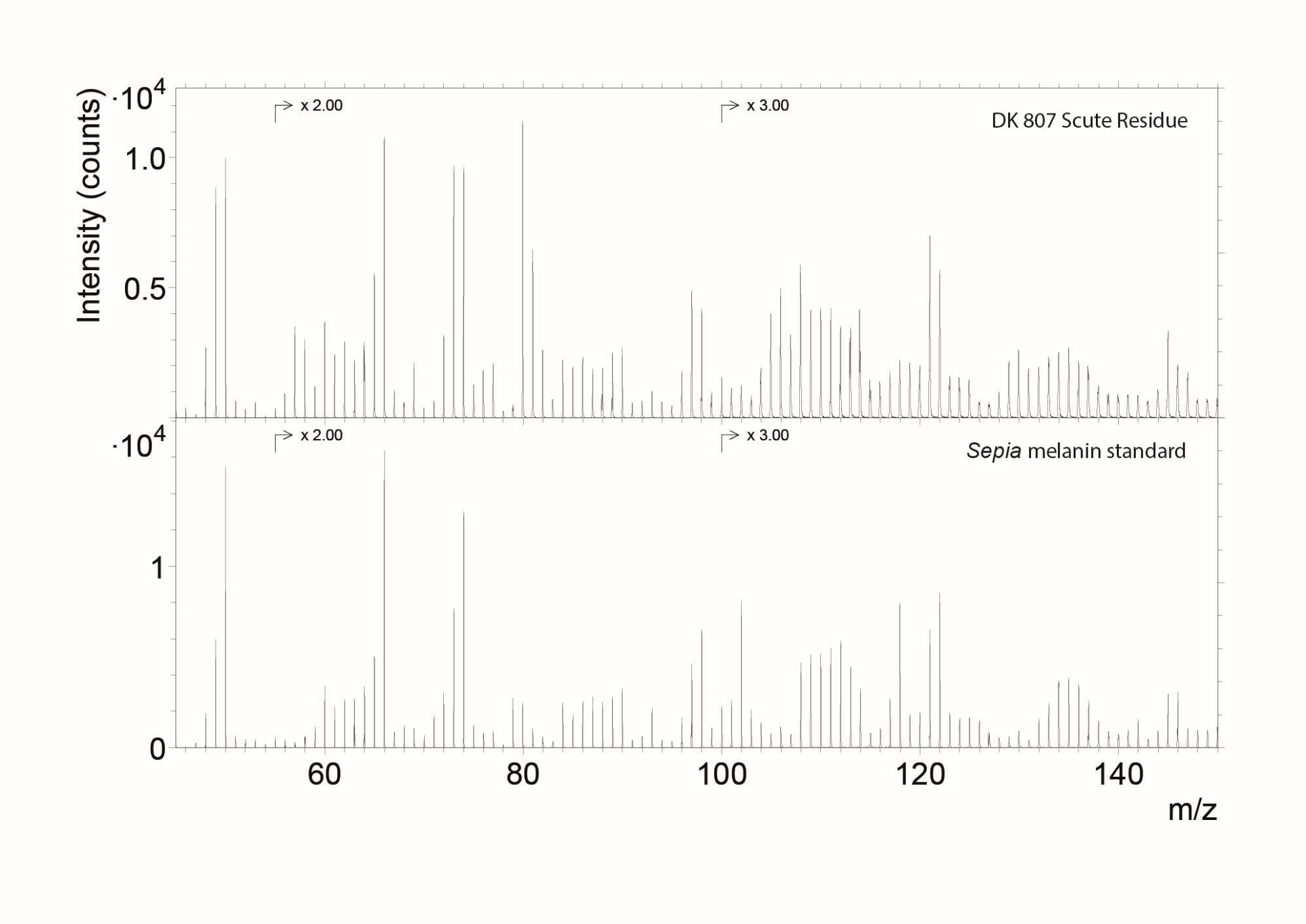


Figure S2. Negative-ion ToF-SIMS spectra of the carbonaceous residue in the scute of DK 807, compared against a *Sepia* eumelanin standard spectrum.


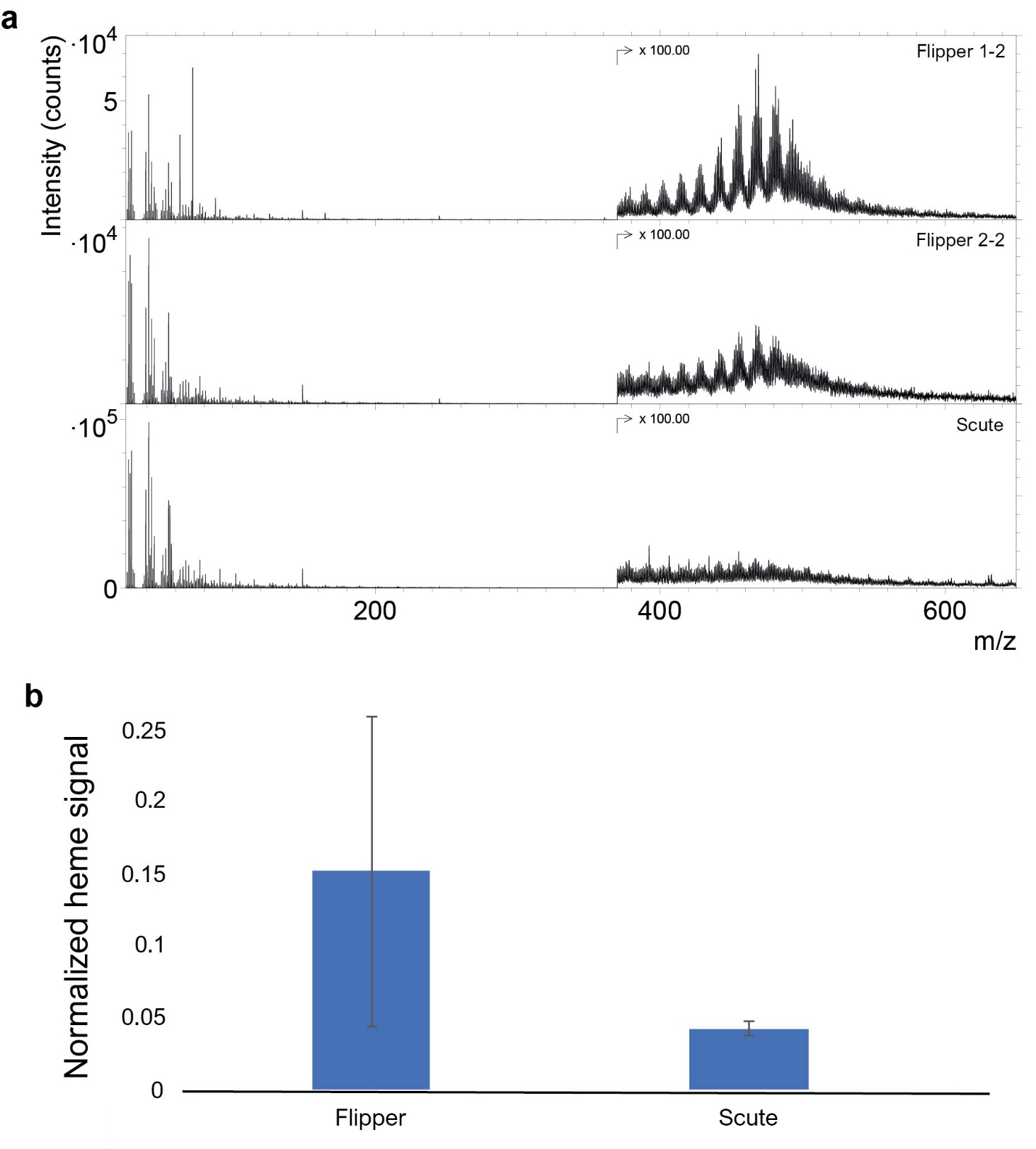
Figure S3. Comparison of heme-related ions in positive ToF-SIMS spectra between the fossil flipper and scute residues. **a**, Representative ToF-SIMS spectra of flipper and scute samples demonstrating higher concentrations of heme in the flipper relative to the scute. **b**, Normalized signal intensities of heme-associated ions (*m/z* 400–520) in positive-ion ToF-SIMS spectra of the fossil flipper and scute residues. The heme signal intensities were normalised to the total signal intensities at *m/z* 25–100, which mainly represent unspecific organic fragment ions. The diagram displays mean values and +/- one standard deviation from 12 flipper and 3 scute spectra.
